# Conference Calendar

**DOI:** 10.1002/cfg.471

**Published:** 2005-04


					Comparative and Functional Genomics
Comp Funct Genom 2005; 6: 181-183.
Published online in Wiley InterScience (www.interscience.wiley.com). DOI: 10.1002/cfg.471
Conference Calendar
July -- September 2005
Animal Models
4th European Zebrafish Development and
Genetics Meeting, July 13-16
http://www.zebrafish-dresden.com/
ESF-FG -- Functional Genomics in Farm
Animals, September 18-20
http://www.functionalgenomics.org.uk/
sections/activitites/2005/Burt/burt.htm
Bioinformatics
GRC -- Bioinformatics: From Predictive
Models to Inference, August 7-12
http://www.grc.org/programs/2005/bioinf.htm
WSEAS -- Grid Computing, August 17-19
http://www.worldses.org/conferences/2005/
corfu/smo/grid/index.html
CHI -- Microarray Data Analysis, August
25-26
http://www.healthtech.com/2005/mda/index.asp
WSEAS -- Applied Informatics and
Communications, September 15-17
http://www.worldses.org/conferences/2005/
malta/aic/index.html
European Conference on Computational
Biology (ECCB), September 28-October 1
http://www.eccb05.org/
Functional Genomics
GRC -- Evolutionary & Ecological Functional
Genomics, July 31-August 5
http://www.grc.org/programs/2005/evolut.htm
CSHL -- Eukaryotic mRNA Processing, August
24-28
http://meetings.cshl.edu/meetings/rnapr05.shtml
SC -- RNAi Europe, September 28-29
http://www.selectbiosciences.com/conferences/
rnaieurope2005/
CSHL -- RNAi, September 28-October 2
http://meetings.cshl.edu/meetings/rnai05.shtml
Human Genomics and Genetics
GRC -- Human Genetics & Genomics, July
24-29
http://www.grc.uri.edu/programs/2005/
humangen.htm
WT -- Population Genomics, September
20-23
http://www.wellcome.ac.uk/doc wtd003175.html
General
SEB -- Main Meeting 2005, July 11-15
http://www.sebiology.org/Meetings/
pageview.asp?S=2&mid=23
WSEAS -- Cellular and Molecular Biology, July
15-17
http://www.worldses.org/conferences/2005/
greece/bio/index.html
BS -- BioScience 2005, July 17-21
http://www.bioscience2005.org/
Copyright  2005 John Wiley & Sons, Ltd.
182 Conference Calendar
GRC -- Chromosome Dynamics, July
31-August 5
http://www.grc.org/programs/2005/
chromdyn.htm
GRC -- Chronobiology, July 31-August 5
http://www.grc.org/programs/2005/chrono.htm
Metabolomics
GRC -- Plant Metabolic Engineering, July
10-15
http://www.grc.uri.edu/programs/2005/
plant.htm
GRC -- Enzymes, Coenzymes & Metabolic
Pathways, July 17-22
http://www.grc.uri.edu/programs/2005/
enzymes.htm
Microbes
GRC -- Microbial Population Biology, July
17-22
http://www.grc.uri.edu/programs/2005/
micropop.htm
IUMS 2005: Microbes in a Changing World, July
23-28
http://www.iums2005.org/iums.asp
GRC -- Applied & Environmental
Microbiology, July 24-29
http://www.grc.uri.edu/programs/2005/
applied.htm
GRC -- Archaea: Ecology, Metabolism &
Molecular Biology, August 14-19
http://www.grc.org/programs/2005/archaea.htm
CSHL -- Yeast Cell Biology, August 16-21
http://meetings.cshl.edu/meetings/yeast05.shtml
CSHL -- Microbial Pathogenesis and Host
Response, September 14-18
http://meetings.cshl.edu/meetings/host05.shtml
Pharmacogenomics/Genomics and
Disease
IBC -- 10th Drug Discovery Technology &
Development, August 8-11
http://www.drugdisc.com/section.asp
GRC -- Malaria, August 21-26
http://www.grc.org/programs/2005/malaria.htm
Drug Discovery, Development and Delivery,
August 30-September 3
http://www.biocom.co.uk/drug2005.htm
ESF-FG -- Functional Genomics and Disease,
September 6-10
http://www.esffg2005.org/
IBC -- Chips to Hits, September 12-15
http://www.chipstohits.com/
WT -- Pharmacogenomics, September 14-18
http://www.wellcome.ac.uk/doc wtd003175.html
CHI -- Toxicity Biomarkers, September 26-27
http://www.healthtech.com/conference-fall.asp
WT -- Neurogenomics, September
28-October 2
http://www.wellcome.ac.uk/doc wtd003175.html
Plants
ASPB -- Plant Biology 2005, July 16-20
http://www.aspb.org/meetings/pb-2005/
GRC -- CO2 Assimilation in Plants: Genome
to Biome, September 11-16
http://www.grc.org/programs/2005/co2.htm
Copyright  2005 John Wiley & Sons, Ltd. Comp Funct Genom 2005; 6: 181-183.
Conference Calendar 183
Proteomics
30th FEBS & 9th IUBMB Congress: The Protein
World, July 2-7
http://www.febs-iubmb-2005.com/
19th Annual Symposium of The Protein Society,
July 30-August 3
http://www.proteinsociety.org/pages/
page00g.htm
HUPO -- 4th Annual World Congress, August
28-September 1
http://www.hupo2005.com/
CHI -- Protein Biomarkers and Mass
Spectrometry for Protein Biomarker
Discovery, September 26-28
http://www.healthtech.com/conference-fall.asp
Systems Biology
Foundations of Systems Biology in Engineering,
August 7-10
http://www.fosbe.org/
ECAL -- Systems Biology Workshop,
September 5
http://www.cmp.uea.ac.uk/sysbio ecal/
Transcriptomics/Regulation
EuroSciCon -- Real Time PCR, July 15
http://www.regonline.co.uk/eventinfo.asp?
EventId=19040
GRC -- Epigenetics, August 7-12
http://www.grc.org/programs/2005/epigen.htm
Biotech Industry
GRC -- Global Aspects Of Technology
Transfer: Biotechnology, September 4-9
http://www.grc.org/programs/2005/global.htm
Bioforum 2005, September 28-29
http://www.bioforum.it/
Key:
ASPB: American Society of Plant Biologists:
http://www.aspb.org/
BS: Biochemical Society:
http://www.biochemistry.org/
CHI: Cambridge Healthtech Institute:
http://www.healthtech.com
CSHL: Cold Spring Harbor Laboratories:
http://meetings.cshl.org/index.htm
ECAL: European Conference on Artificial Life:
http://www.ecal2005.org/
ESF-FG: European Science Foundation Programme
in Functional Genomics:
http://www.functionalgenomics.org.uk/
EuroSciCon: http://www.euroscicon.com/
FEBS: Federation of European Biochemical Soci-
eties: http://www.febs.org/
GRC: Gordon Research Conferences:
http://www.grc.uri.edu/
HUPO: Human Proteome Organisation:
http://www.hupo.org
IBC: IBC Conferences: http://www.ibc-lifesci.com
IUBMB: International Union of Biochemistry and
Molecular Biology: http://www.iubmb.unibe.ch/
IUMS: International Union of Microbiological
Societies: http://www.iums.org/
SC: Select Conferences:
http://www.selectbiosciences.com
SEB: Society for Experimental Biology:
http://www.sebiology.org/
WSEAS: World Scientific and Engineering
Academy and Society: http://www.worldses.org
WT: Wellcome Trust: http://www.wellcome.
ac.uk/
The Conference Calendar is a listing of forthcoming conferences of interest to our readership. We provide
the title, dates and web address of each conference, to enable readers to find out more information.
Inclusion does not imply endorsement by the journal. If you know of a relevant conference, please
contact the Managing Editor.
Copyright  2005 John Wiley & Sons, Ltd. Comp Funct Genom 2005; 6: 181-183.

				

